# Interferon-beta inhibits human glioma stem cell growth by modulating immune response and cell cycle related signaling pathways

**DOI:** 10.1186/s13619-022-00123-w

**Published:** 2022-07-02

**Authors:** Xin-Xin Han, Shengkai Jin, Li-Ming Yu, Min Wang, Xin-Yu Hu, Dai-Yu Hu, Jie Ren, Meng-Han Zhang, Wei Huang, Jia-Jia Deng, Qing-Qing Chen, Zhengliang Gao, Hua He, Chunhui Cai

**Affiliations:** 1 Shanghai Key Laboratory of Craniomaxillofacial Development and Diseases, Shanghai Stomatological Hospital & School of Stomatology, Fudan University, Shanghai, China; 2 Institute of Photomedicine, Shanghai Skin Disease Hospital, Tongji University School of Medicine, Shanghai, China; 3 School of Medicine, Jiaxing University, Jiaxing, China; 4 Tongji University Cancer Center, Shanghai Tenth People’s Hospital, School of Medicine, Tongji University, Shanghai, China; 5 Department of Neurosurgery, Changzheng Hospital, Second Military Medical University, Shanghai, China; 6 Department of Neurosurgery, Third Affiliated Hospital of Second Military Medical University, Shanghai, China

**Keywords:** Glioma stem cells, IFN-β, Neural stem cell, Cell cycle, Immune response

## Abstract

Malignant Glioma is characterized by strong self-renewal potential and immature differentiation potential. The main reason is that malignant glioma holds key cluster cells, glioma stem cells (GSCs). GSCs contribute to tumorigenesis, tumor progression, recurrence, and treatment resistance. Interferon-beta (IFN-β) is well known for its anti-proliferative efficacy in diverse cancers. IFN-β also displayed potent antitumor effects in malignant glioma. IFN-β affect both GSCs and Neural stem cells (NSCs) in the treatment of gliomas. However, the functional comparison, similar or different effects of IFN-β on GSCs and NSCs are rarely reported. Here, we studied the similarities and differences of the responses to IFN-β between human GSCs and normal NSCs. We found that IFN-β preferentially inhibited GSCs over NSCs. The cell body and nucleus size of GSCs increased after IFN-β treatment, and the genomic analysis revealed the enrichment of the upregulated immune response, cell adhesion genes and down regulated cell cycle, ribosome pathways. Several typical cyclin genes, including cyclin A2 (*CCNA2)*, cyclin B1 (*CCNB1)*, cyclin B2 (*CCNB2),* and cyclin D1 (*CCND1)*, were significantly downregulated in GSCs after IFN-β stimulation. We also found that continuous IFN-β stimulation after passage further enhanced the inhibitory effect. Our study revealed how genetic diversity resulted in differential effects in response to IFN-β treatment. These results may contribute to improve the applications of IFN-β in anti-cancer immunotherapy. In addition, these results may also help to design more effective pharmacological strategies to target cancer stem cells while protecting normal neural stem cells.

## Background

Malignant glioma is highly aggressive and represents the most common primary brain tumors. The overall survival period is typically less than 15 months. Even with regular therapy, including surgical resection and chemoradiation, tumor recurrence appears to be inevitable (Ostrom et al. 2016). One year after diagnosis, approximately 70% of malignant glioma patients will facing the disease progression (Stupp et al. 2005). Thus, the use of personalized drugs that target molecular receptors and immunotherapy have been viewed as promising new options for glioma treatment (Davis 2016).

Malignant glioma is heterogeneous, with multiple epigenetic and genetic variations identified in associated tumor cells. The intrinsic, aggressive behavior of malignant glioma has also been shown to depend on the complex tumor microenvironment (TME). Malignant glioma and their TME consist of GSCs, mature neural cells (oligodendrocytes, astrocytes, microglia, and ependymal cells), and some immune cells etc. (Abels et al. 2019; Broekman et al. 2018).

The identification of cancer stem cells in malignant glioma are first reported in 2002 (Ignatova et al. 2002). Several groups isolate and characterize stem-like cancer cells in glioma (Galli et al. 2004; Hemmati et al. 2003; Ignatova et al. 2002), which lead to the realization that GSCs are resistant to chemotherapy and radiotherapy (Bao et al. 2006; Chen et al. 2012). Some groups try to find the genes which regulate GSCs maintenance and glioma progression (Herrmann et al. 2020; Hu et al. 2016; Huang et al. 2021; Kim et al. 2021; Taga and Tabu 2020; Venkatesh et al. 2015; Wang et al. 2021). Inspire of so many struggles, the prognosis of GBM has not enhanced in the past decade (Wang et al. 2021). At the same time, the underlying mechanisms of GSCs survival after treatment remain unclear.

Interferon (IFN) factors are pleiotropic cytokines, it can be categorized into 3 classes. Type I IFNs include approximately 20 members. Human IFNs induce the Janus kinase–signal transducer (JAK) and activator of transcription (−STAT) cascade by binding to the IFN-α/β receptors (IFNARs), IFNAR1 and IFNAR2 (Lohmann et al. 2020; Platanias 2005). IFN-β signaling had been proved to inhibit cell proliferation in many types of cancer cells (Borden et al. 2000; Mizuno and Yoshida 1998; Natsume et al. 1999; Natsume et al. 2000; Yagi et al. 1994). During malignant glioma treatment, at least two aspects of neural stem cells (NSCs) are related to GSCs. On the one hand, mutated NSCs are considered to be the initiation cells of glioma (Alcantara Llaguno et al. 2019; Tian et al. 2020; Wang et al. 2021). On the other hand, normal NSCs are considered to have the ability to move towards GSCs, and can be used as carriers for the treatment of gliomas (Kendall et al. 2008; Schmidt et al. 2005). The human F3 NSCs cell line has been used in multiple studies to perform NSC-based gene therapy, delivering both IFN-β and cytosine deaminase (CD)/5-fluorocytosine (5-FC) prodrugs to glioma cells (Dickson et al. 2007; Kim et al. 2006; Shimato et al. 2007). Although IFN-β has been generally employed as a clinical treatment, whole-transcriptome analyses examining the effects of IFN-β stimulation in GSCs and NSCs are still rare. A systematic understanding of the genetic variations that occur following IFN-β treatments can provide additional evidence for the optimization of IFN-β-associated gene therapy for malignant glioma treatment in clinical trials.

In the present study, we used various doses of IFN-β to separately treat human GSCs (hGSCs) and human NSCs (hNSCs). Both morphological and genetic alterations were carefully observed and analyzed. We found that IFN-β increased the cell and nuclear size of hGSCs. The number of sphere-like cells observed in hGSC populations was reduced, both during short-term treatments and under conditions of continuous stimulation. However, INF-β did not appear to have the same or similar effects on hNSCs. Genomic analysis was performed to identify genes with expression changes in hGSCs but not in hNSCs. Immune response and cell adhesion genes were upregulated by IFN-β treatment, whereas the expression levels of cell cycle and ribosome genes were strongly reduced, which was consistent with our observations of the changed cell morphology. We observed that IFN-β preferentially restrained hGSCs rather than hNSCs. A few cyclin genes, including *CCNA2*, *CCNB1/2* and *CCND1*, downregulated in hGSCs after IFN-β treatment. Our exploration may facilitate to design new and more effective pharmacological strategies for killing hGSCs while protecting hNSCs during glioma treatment.

## Results

### IFN-β inhibits the growth of hGSCs

**Fig. 1:**
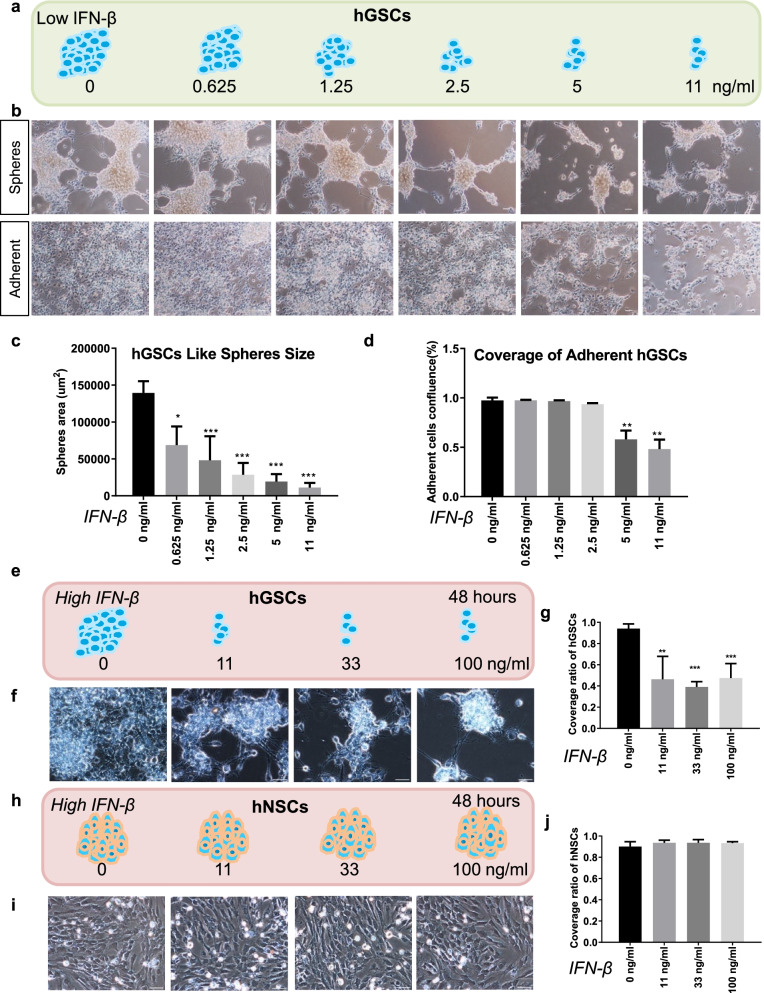
The growth of hGCSs but not hNSCs is inhibited by IFN-β. **a** A schematic representation showing hGCSs treated with basic media containing 10 ng/mL fibroblast growth factor (FGF), and different concentrations of IFN-β: 0, 0.625, 1.25, 2.5, 5, and 11 ng/mL. **b** The sphere-like hGCSs were inhibited, and more adherent hGCSs were observed after the IFN-β treatment. **c** IFN-β treatment resulted in smaller single sphere-like cells than control cells, and the sizes of the cell spheres are concentration-dependent within a certain range of IFN-β concentrations. **d** Coverage rate of adherent hGCSs decreased significantly with 5 ng/mL and 11 ng/mL IFN-β treatments. **e** Schematic representations of hGCSs treated with high concentrations of IFN-β. **f** High-concentration IFN-β also inhibits hGCS growth. **g** The coverage rate of hGCSs decreased after treatment with 11 ng/mL, 33 ng/mL, and 100 ng/mL IFN-β. **h** Schematic representation of hNSCs treated with high-concentration IFN-β. **i** High-concentration IFN-β does not inhibit hNSC growth. **j** The coverage rate of hNSCs does not decrease following treatment with 11 ng/mL, 33 ng/mL, and 100 ng/mL IFN-β. Quantitative data measured using ImageJ. Data are presented as the mean ± SD. Student’s t-test. ∗*p* < 0.05, ∗∗*p* < 0.01, ∗∗∗*p* < 0.001

To detect the functional role of IFN-β in hGSCs, we first used IFN-β, at concentrations of 0, 0.625, 1.25, 2.5, 5, and 11 ng/mL, to treat the hGSCs (Fig. 1a). hGSCs typically display two types of cell morphologies: sphere-like hGSCs and adherent hGSCs (Fig. 1b). We quantified the area sizes of the sphere-like hGSC and the coverage of adherent hGSCs to examine the effects of IFN-β on cell growth. The sizes of the sphere-like hGSCs significantly decreased, even with the lowest treatment of 0.625 ng/mL IFN-β (Fig. 1c). However, no changes in the coverage of adherent hGSCs were observed until the treatment concentration reached 5 ng/mL IFN-β (Fig. 1d). These observations suggested that adherent hGSCs have a higher tolerance against IFN-β treatment than sphere-like cells. We repeated the treatment assay using higher IFN-β concentrations, including 11, 33, and 100 ng/mL, on both hGSCs and hNSCs (Fig. 1e, h). High dose of IFN-β also inhibited hGSC growth, but no significant morphological changes were observed with treated hNSCs (Fig. 1f, i). The quantitative analysis of cell coverage supported our visual observations (Fig. 1g, j). In conclusion, both low-dose (0.625 ng/mL) and high-dose (up to 33 ng/mL) IFN-β treatments were able to block cell growth in hGSCs, without affecting hNSCs.

### The nuclear size of hGSCs was increased by IFN-β

**Fig. 2:**
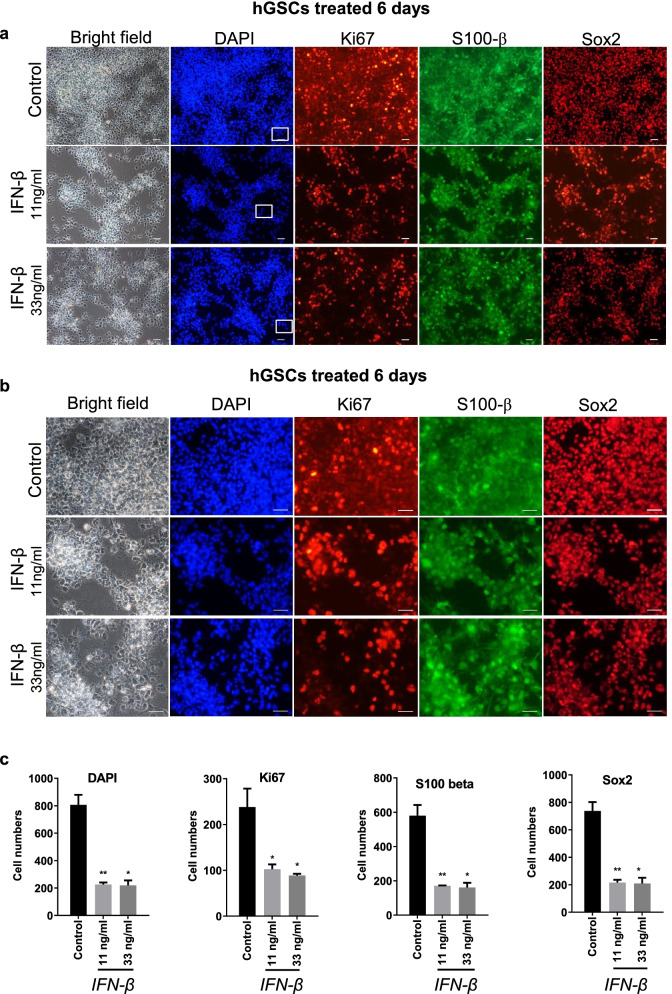
IFN-β treatment resulted in decreased numbers of Ki67-, S100-β-, and Sox2-positive cells. **a** Representative images of hGCSs immunostained for Ki67, S100-β, and Sox2, with DAPI nuclear stain. Three groups, control, 11 ng/mL, and 33 ng/mL IFN-β treatment groups, were collected after 6 days of treatment (scale bar, 50 μm; white frames indicate the next part of the figure). **b** Representative images from (A), at higher magnification, using the same three groups: control, 11 ng/mL, and 33 ng/mL IFN-β treatment groups. **c** Quantitative analysis of Ki67, S100-β, Sox2, and DAPI nuclear stain in hGCSs after IFN-β stimulation, as measured by ImageJ. Data are presented as the mean ± SD. Student’s t-test. ∗*p* < 0.05, ∗∗∗*p* < 0.001

**Fig. 3:**
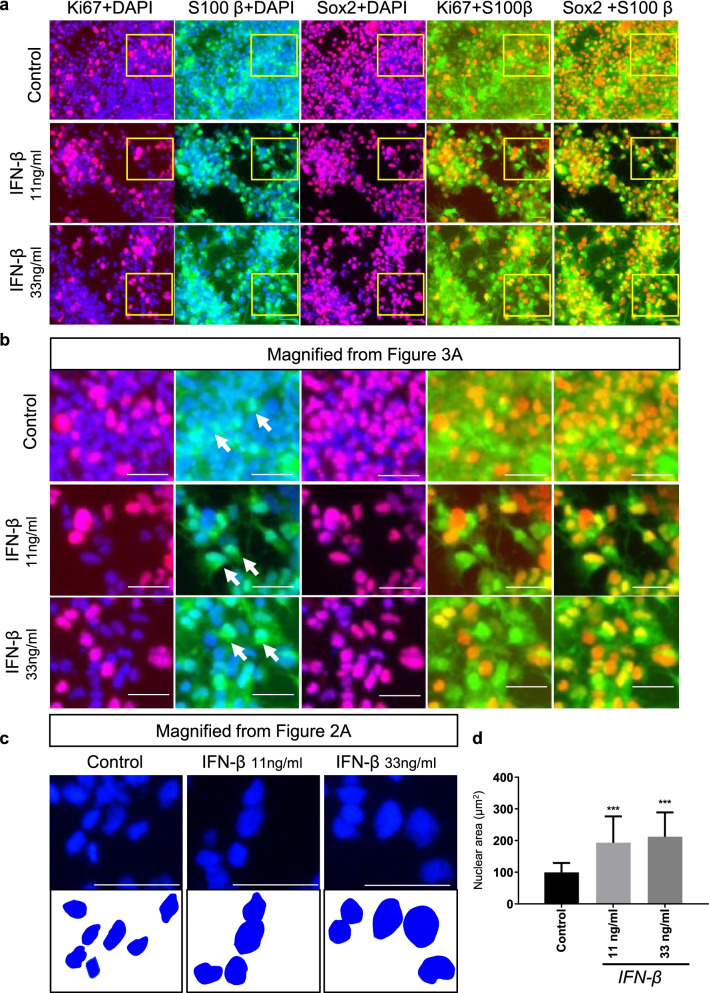
Merged images showing the relative expression level of S100-β and the increased size of the cell nucleus after IFN-β stimulation. **a** Representative overlapping images showing staining for Ki67/DAPI, S100-β/DAPI, Sox2/DAPI, Ki67/S100-β, and Sox2/S100-β in hGCSs. Three groups, control, 11 ng/mL, and 33 ng/mL IFN-β treatment groups (yellow frames indicate enlarged areas). **b** Representative images from (a) at higher magnification. White arrows indicate S100-β expression. Enlarged area (scale bar, 50 μm). **c** Representative images of DAPI staining and a schematic diagram of the nucleus. **d** Quantitative analysis of nuclear size, as measured using ImageJ. Scale bar, 50 μm. Data are presented as the mean ± SD. Student’s t-test. ∗∗∗*p* < 0.001

We next performed immunostaining to detect the expression of Ki67, S100-β, and Sox2 in hGSCs after 6 days of treatment of IFN-β at both low (11 ng/mL) and high (33 ng/mL) doses. Similar to our morphological observations, the number of Ki67-positive hGSCs decreased after IFN-β treatment (Fig. 2a, b), as did the numbers of S100-β- and Sox2-positive cells (Fig. 2a, b). The quantitative assessment of Ki67, S100-β, Sox2, and DAPI nuclear stain in hGSCs following IFN-β stimulation can be seen in Fig. 2c. Because the total cell number decreased, the ratio of Ki67 to DAPI staining did not show a difference between the control group and the IFN-β-treated group. Overlapping images showed that the relative expression level of S100-β increased significantly after IFN-β stimulation (Fig. 3a). IFN-β might represent an effective agent for controlling cell differentiation in hGSCs (Yamamuro et al. 2015). S100-β has long been considered a biomarker for astrocytes (Castets et al. 1997). Recently, some studies have also identified the high expression levels of S100-β with malignant tumors (Imbalzano et al. 2020). The increased S100-β expression observed in hGSCs following IFN-β stimulation agrees with previous reports. The higher magnification image revealed the nuclear status (Fig. 3b). Representative images of DAPI staining and the schematic diagram of the nucleus revealed significant nuclear enlargement after IFN-β stimulation (Fig. 3c). The nucleus size was quantified using ImageJ, which revealed that the size of the nucleus increased in a dose-dependent manner following IFN-β treatment (Fig. 3d).

### The cell growth and size of hGSCs was reduced by repetitive stimulation treatment with IFN-β

**Fig. 4:**
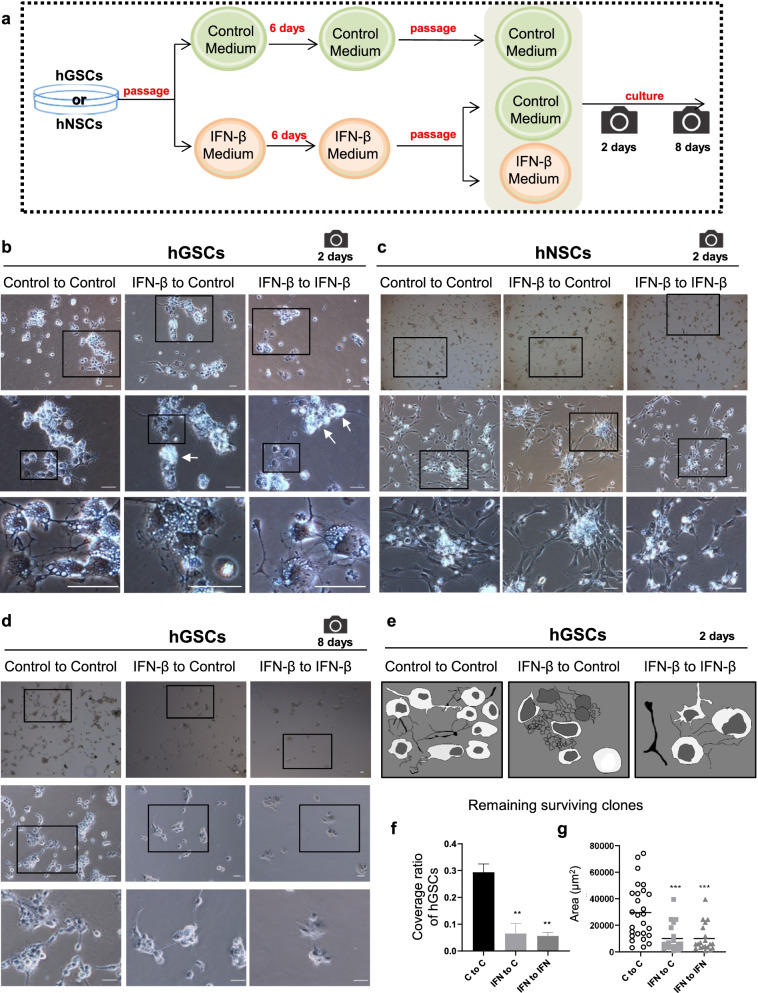
Continuous IFN-β stimulation can enhance the inhibitory effects on hGCS growth, which differs from the effects on hNSCs. **a** Flow chart indicating the continuous stimulation timeline and imaging time points. The strategy of passage → stimulation → waiting → re-passage → stimulation → time point photography was adopted. **b** Representative hGCS images after continuous stimulation, re-passage, and stimulation, day 2. Three groups: control to control, IFN-β to control, and IFN-β to IFN-β (black frames indicate enlarged areas). **c** Representative hNSC pictures after continuous stimulation, re-passage, and stimulation, day 2. Three groups: control to control, IFN-β to control, and IFN-β to IFN-β (black frames indicate the enlarged area). **d** Representative hGCS images after continuous stimulation at re-passage, day 8. **e** Schematic diagram of hGCSs and hNSCs after re-passage and stimulation, day 2. **f**-**g** Quantitative analysis of hGCSs, including coverage rate (F) and single-clone area (G), of the surviving clones, as measured by ImageJ. Scale bar, 50 μm. Data are presented as the mean ± SD. Student’s t-test. ∗∗*p* < 0.01, ∗∗∗*p* < 0.001

Clinically, IFN-β is administered over a long time course. Thus, we examined the effects of continuous IFN-β stimulation. Both hGSCs and hNSCs were cultured in either control medium or IFN-β medium for one generation (6 days), and then the control cells were passaged into control medium, whereas IFN-β-treated cells were separately passaged into both control medium and IFN-β medium (Fig. 4a), generating three groups of cells: control to control, IFN-β to control, and IFN-β to IFN-β. On day 2, the number of sphere-like hGSCs in the IFN-β to IFN-β group decreased compared with the numbers observed in the control to control group (Fig. 4b). The hGSCs conditions in IFN-β to control group showed more sphere-like cells than IFN-β to IFN-β group but fewer than observed in the control to control group. Although no significant effects were observed among hNSCs following short-term IFN-β stimulation (Fig. 1i), continuous IFN-β stimulation resulted in decreased cell growth (Fig. 4c). We observed these groups of cells again on day 8, which revealed very few surviving sphere-like cells in hGSCs within the IFN-β to IFN-β group (Fig. 4d). The enlargement of both the cell body and the nuclear size was also observed in this group (Fig. 4e). The hGSCs in the IFN-β to IFN-β group displayed an oligodendrocyte-like morphology, with a long and massive synapse (Fig. 4e). Quantitative analysis of hGSCs, including the coverage rate (Fig. 4f) and the single clone area (Fig. 4g), was performed on the surviving clones, which revealed significant reductions associated with IFN-β treatment.

### Different gene responses in hGSCs and hNSCs after IFN-β treatment

**Fig. 5:**
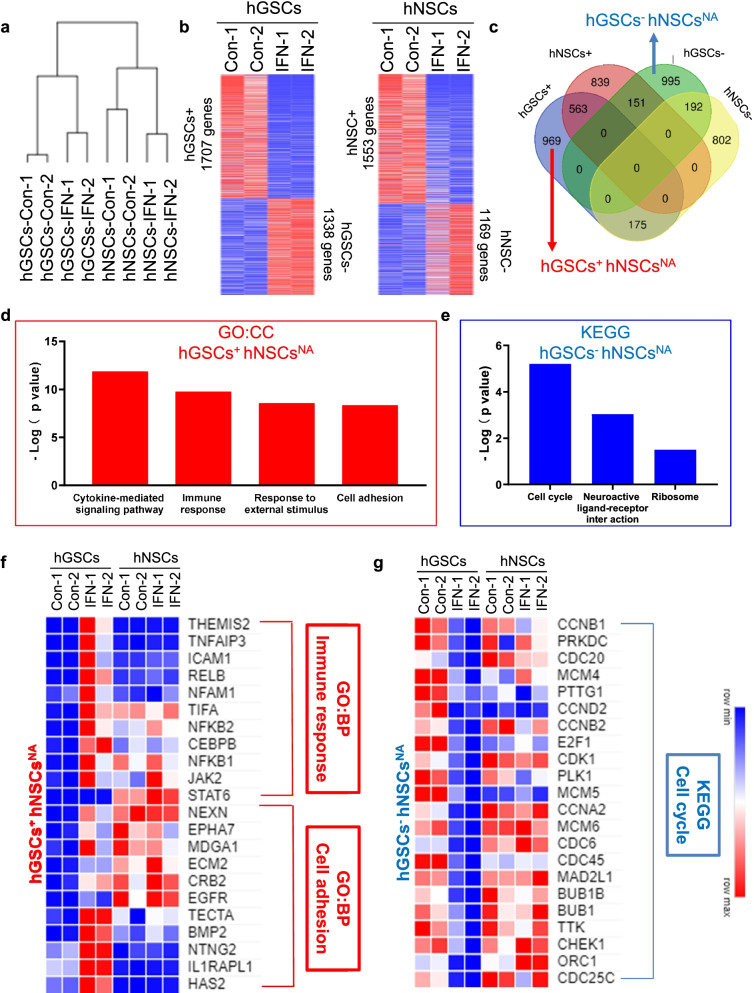
Genomic changes in hGCSs after IFN-β treatment. **a** Gene clustering from control (Con-1 and Con-2) and IFN-β treatment (IFN-1 and IFN-2) samples for the hGCSs/hNSCs RNA sequencing results. **b** Identification of significantly upregulated and downregulated genes as hGCS^+^ (1707 genes), hGCS^−^ (1338 genes), hNSC^+^ (1553 genes), and hNSC^−^ (1169 genes). **c** The Venn diagram shows the overlap among the 4 gene lists identified in (B) to identify hGCS−hNSC^NA^ (995 genes) and hGCS^+^hNSC^NA^ (969 genes) lists. **d** Biological process (BP) items in the Gene Ontology analysis of the hGCS^+^hNSC^NA^ gene list. **e** KEGG analysis of the hGCS^−^hNSC^NA^ gene list. **f** Expression patterns of immune response and cell adherent genes in hGCSs and hNSCs. **g** Expression patterns of cell cycle genes in hGCSs and hNSCs

To better understand the molecular mechanism associated with the IFN-β treatment effects observed in hGSCs and hNSCs, we performed RNA sequencing on samples treated with or without IFN-β in both hGSCs and hNSCs. Gene clustering analysis indicated the high quality of our whole-transcriptome data (Fig. 5a). The analysis of different genes expression (DEGs) was used to identify genes that were up- and downregulated following IFN-β stimulation, resulting in 1707 and 1338 genes designated as hGSC^+^ and hGSC^−^ genes, respectively, whereas 1553 and 1169 genes were respectively designated as hNSC^+^ and hNSC^−^ genes (Fig. 5b). The integrative analysis of these four groups resulted in the identification of 995 genes characterized as hGSC^−^hNSC^NA^ (genes only downregulated in hGSCs but with no change in hNSCs) and 969 genes characterized as hGSC^+^hNSC^NA^ (genes only upregulated in hGSCs but with no change in hNSCs) (Fig. 5c). Then, we performed Kyoto Encyclopedia of Genes and Genomes (KEGG) and Gene ontology (GO) analyses on these two groups to further explore the potential downstream mechanisms associated with the response to IFN-β. The GO biological process (BP) analysis revealed that hGSC^+^hNSC^NA^ genes were primarily enriched in cytokine-mediated signaling, immune response, response to external stimulus, and cell adhesion pathways (Fig. 5d). The KEGG analysis revealed that hGSC−hNSC^NA^ genes were enriched in the cell cycle and ribosome pathways (Fig. 5e). Our whole-transcriptome analysis results agreed with our previous morphological observations, supporting increased cell adherence and decreased cell growth. The expression patterns of the immune response and cell adhesion genes were displayed as heatmaps for both hGSCs and hNSCs (Fig. 5f). Consistent with known IFN-β downstream pathways, *JAK2*, *STAT6*, and *NFKB1*/2 were identified within the immune response pathways (Pfeffer et al. 2004; Platanias 2005; Yang et al. 2000). We also explored the detailed expression patterns of 22 genes in the cell cycle pathway (Fig. 5g). Typical cell cycle-related genes, such as *CCNA2*, *CCNB1*, *CCNB2,* and *CCND1,* were significantly downregulated following IFN-β stimulation in hGSCs but remained unchanged in hNSCs.

### IFN-β decreased cell proliferation in hGSCs

**Fig. 6:**
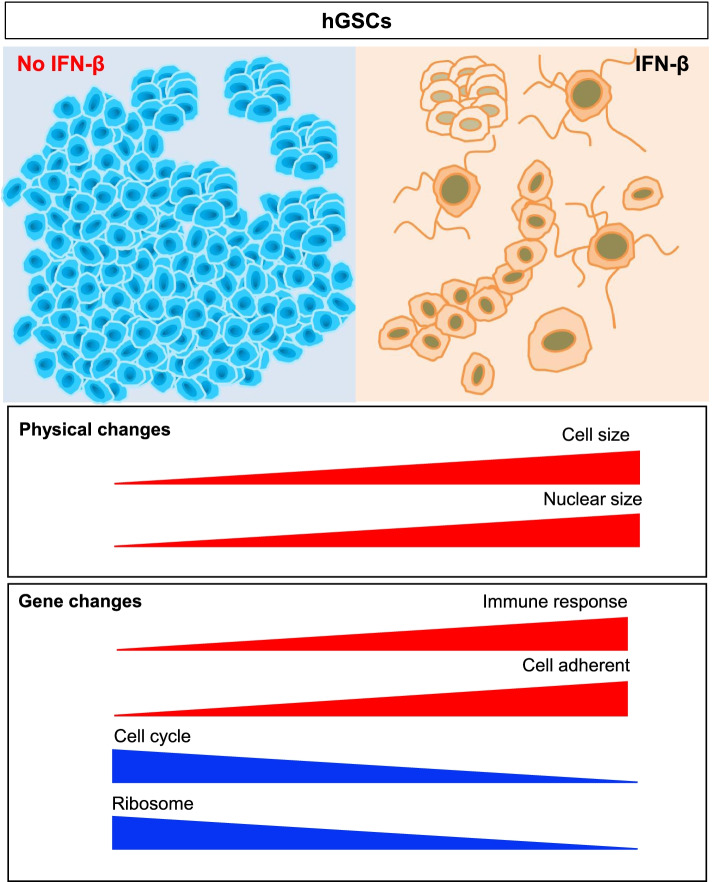
IFN-β reduced cell proliferation in hGCSs. The ability to form spheres was inhibited by IFN-β. The cell and nuclear sizes of hGCSs increased after IFN-β treatment. On a gene level, significant upregulation was observed among immune response and cell adherence-related genes. Cell cycle and ribosome-related genes decreased

IFN-β decreased hGSCs cell growth on both the morphological and genetic levels. Cell cycle-related genes were significantly downregulated after IFN-β stimulation (Figs. 5g and 6). The number of sphere-like cells also reduced significantly following IFN-β treatment, and both cell body and nuclear size increased. Simultaneously, genes associated with cell adhesion were upregulated in hGSCs, which supported the observed reductions in sphere-like cells and the enlargement of the cell nucleus. We also observed multiple synapses in hGSCs under conditions of continuous stimulation with IFN-β, and this morphological change may be associated with changes in gene expression in the immune response pathway.

## Discussion

After stimulation of IFN-β, both the cell size and nuclear size of hGSCs increased. The increasement of nuclear size may directly correlated with the whole cell size enlargement. In both budding and fission yeasts, cells maintain a stable nuclear volume to cell volume ration at around 8% (Jorgensen et al. 2007; Neumann and Nurse 2007). Nuclear transfer experiment with Hela cells double confirmed that it is the cell size instead of DNA content to determine nuclear size (Cantwell and Nurse 2019). The cell size control mechanism is complex but important to maintain body homeostasis. The size theory suggested that cell division is based on a critical cell size (Cadart et al. 2018; Chien et al. 2012; Turner et al. 2012). In the yeast and bacteria studies, they indicated that large cells tend to proliferate faster than smaller ones (Chien et al. 2012; Turner et al. 2012). In mammalian cells, research of cell size distributions in populations of lymphoblasts shown support of the size theory. At the same time, researchers concluded that mammalian cells may have an intrinsic mechanism for cell size maintenance which is independent of cell cycle or age (Cadart et al. 2018; Son et al. 2012). In our data, the increased cell size did not correlate with faster cell growth. On the other side, less cell number and decreased cell cycle related gene expression have been observed after IFN-β stimulation in hGSCs. Similar phenomenon had been reported in mesenchymal stem cells (MSCs) before. MSCs had been separated as smaller and bigger cells (i.e., > 8-μm and < 8-μm) by pore transwell inserts, the sieved cells (< 8-μm) shown higher proliferative probability than un-sieved cells (> 8-μm) (Corradetti et al. 2011). In another study, the cellular thickness of MSCs had been measured with atomic force microscopy. Their results also suggested that small cells had high proliferative activity while large and flat cells have low proliferative activity (Katsube et al. 2008). The correlation between cancer stem cells (CSCs) and cell size had been investigated in prostate cancer cell lines PC3 by cell cytometry sorting technique (Li et al. 2015). The functional roles test shown that large PC3 cells (≧ 20 or 30 μm) demonstrated lower clonal capacity and less tumorigenic ability than small PC3 cells (< 10 μm) (Li et al. 2015). However, it is still not clear which type of mechanisms coordinate cell growth and proliferation in metazoan cells (Li et al. 2015). Cell size enlargement and decreased cell proliferative ability in our study support the previous correlation of cancer stem cells and their sizes, further genomic analysis with different sizes of cancer stem cells may help us better understand the molecular mechanisms.

Our results indicated that IFN-β affected hGSCs rather than hNSCs. hNSCs are considered to be the most likely initiation cells of malignant glioma (Alcantara Llaguno et al. 2019; Tian et al. 2020; Wang et al. 2021). On the other hand, hNSCs have been considered efficient vehicles for the delivery of anti-cancer agents to tumor sites during therapeutic applications (Tang et al. 2017; Yamazoe et al. 2015). IFN-β has been found to exert antiproliferative effects in many cancer cell types (Ito et al. 2010; Sims et al. 2008). In glioma cell lines, IFN-β can inhibit cell cycle S-phase to decrease cell proliferation and progression (Garrison et al. 1996). However, the underlying molecular mechanisms responsible for the subsequent differences observed following IFN-β treatment between cancer stem cells and normal NSCs remain unclear. Microarray-based gene expression profiling performed in three glioma cell lines and primary B-cells following IFN-β treatment was reported in 2014 (Happold et al. 2014; Khsheibun et al. 2014). In primary B-cells, several novel IFN-β response genes were identified including *NEXN*, *HAPLN3*, *DDX60L,* and *IGFBP4* (Khsheibun et al. 2014). In this study, RNA sequencing was performed to reveal the molecular mechanisms associated with the response to IFN-β. We explored potential IFN-β response genes in glioma cells by performing DEG analysis between hGSCs and hNSCs. A total of 969 genes were identified as IFN-β-upregulated genes, whereas 995 genes were identified as IFN-β-downregulated genes in hGSCs compared with hNSCs. This large number of genes provides multiple opportunities to identify potential therapeutic targets that can be combined with IFN-β treatments for clinical applications.

A previous study indicated that IFN-β could induce spherogenicity in hGSCs (Happold et al. 2014). In our study, we noticed a significant reduction in sphere-like cells following IFN-β stimulation. The Gene Ontology (GO) analysis results indicated enrichment in cell adhesion genes, providing strong support for a functional role of IFN-β in cancer stem cell morphology. *NEXN* encodes nexilin, which acts as a linker protein for the cytoskeleton and affects focal adhesion junctions (Ohtsuka et al. 1998). *NEXN* was detected as a novel IFN-β response gene in multiple sclerosis (Khsheibun et al. 2014). In the whole-transcriptome analysis following IFN-β treatment, *NEXN* was identified as a top gene in the enriched cell adhesion pathway in hGSCs after IFN-β treatment. Therefore, *NEXN* may also represent a novel IFN-β response gene in hGSCs.

The genes that were downregulated by IFN-β treatment were primarily enriched in cell cycle pathways, which also provides evidence to support the previously identified inhibitory role of IFN-β in cancer cells (Garrison et al. 1996; Ito et al. 2010; Sims et al. 2008). A total of 22 cell cycle-related genes were found to be downregulated in hGSCs, but no significant differences were observed in hNSCs. We suggested that these 22 genes may play irreplaceable roles in response to IFN-β treatment in hGSCs. Recently, one study attempted to combine IFN-β treatment together with a novel, effective, cyclin-dependent kinase (CDK) inhibitor (TG02), which is used clinically to treat GBM (Le Rhun et al. 2019; Su et al. 2018; Wu et al. 2017), resulting in synergistic functions in human glioma models (Lohmann et al. 2020). In our data, *CDK1* was identified in the genomic analysis. Many other well-known cell cycle-related genes, such as the cyclin family genes (*CCNB1*, *CCND2*, *CCNB2,* and *CCNA2*), and MCM family genes (*MCM4*, *MCM5,* and *MCM6*), were also identified. These results may facilitate the development of more efficient therapeutic options by guiding the selection of useful anti-tumor drugs.

In our study, we included both hGSCs and hNSCs from previous studies (Han et al. 2017; Han et al. 2021). For each cell type, we include two cell lines for RNA sequencing analysis. Around 1500 genes regulated by IFN-β have been successfully identified with DGE analysis (Fold change > 2) as shown in Fig. 5b. Then we draw venn diagram to help us better understand the distribution of these genes. To better understand the specific roles of IFN-β on hGSCs rather than hNSCs, we only focus on 969 genes upregulated in hGSCs (hGSC^+^hNSC^NA^) and 995 genes downregulated in hGSCs (hGSC^−^hNSC^NA^) as shown in Fig. 5c. Together with the morphological modification in hGSCs under IFN-β stimulation, we narrow down our target on cell cycle and cell adhesion pathways with KEGG and GO analysis. Further transcriptional analysis with multiple cell lines or primary tissues from these two different cell types may help us to deeply understand the underneath molecular mechanism. Also, the investigation of different dosages of IFN-β stimulation combined with regular chemotherapy drug, such as Temozolomide will be more persuasive for clinical consideration.

## Conclusions

In summary, our study established the inhibition effects of IFN-β in hGSCs rather than in hNSCs. Additional morphological details were observed following IFN-β stimulation, such as larger cell bodies and nuclear sizes, fewer sphere-like cells, and more oligodendrocyte-like synapses. The subsequent transcriptional analysis using RNA sequencing was highly consistent with our morphological observations of hGSCs. The enrichment of genes involved in the cell cycle and cell adhesion pathways supported the observed reduction in cell growth and the morphological changes associated with IFN-β treatment. In addition, our exploration of the genetic modification that occurs after IFN-β treatment in both hGSCs and hNSCs may benefit the design of new and more effective pharmacological strategies for GBM treatment.

## Methods

### Cell culture

hGSCs line was established from surgical specimens (Han et al. 2021). Surgical specimens were gotten strictly according to Ethics Committee permission. Informed consents were introduced to glioma patients. All surgical specimens were donated with patients’ permission. Tumor samples were delivered to the laboratory immediately after surgery for subsequently procedures. Briefly, after surgery, surgical specimens were collected for primary culture. The specimens were washed with 1× Hank’s Balanced Salt Solution (HBSS, Gibco) at least six times. Then, the specimens were cut into small pieces, and the tissue fragments were transferred into 15 mL centrifuge tubes containing 1 U/mL Dispase II (Roche) in 3 mL Dulbecco’s Modified Eagle Medium (DMEM)/F12 (Gibco) and kept in water-bath at 37 °C for 30 minutes to allow digestion. After digestion, the specimens were centrifuged at 1000×g for 3 minutes. The supernatant was discarded, and the tissues were resuspended with 3 mL DMEM/F12, followed by centrifugation. Finally, the precipitate was resuspended in growth medium (DMEM/F12 supplemented with N-2, B-27, GlutaMAX, bFGF, EGF, heparin and penicillin-streptomycin) for daily culture. All the work concentration of FGF, EGF and heparin were 20 ng/Ml, N-2, GlutaMax and penicillin-streptomycin were 100X while B-27 were 50X in the culture medium.

hNSCs line was generated from human embryonic stem cells (hESCs) (Han et al. 2017). Brief description, StemPro Accutase (Thermo Fisher) was used to digest the hESCs for 20 min at 37 °C. The cells were plated onto gelatin-coated plates for 1 hour at 37 °C. Because hESCs remain suspended, whereas mouse embryonic fibroblast (MEF) cells are adherent and separate hESCs from MEFs. The non-adherent hESCs were washed and seeded on Matrigel-precoated dishes in MEF-conditioned medium. Then we changed the medium to remove the ROCK inhibitor after 24 hours. Single adherent hESCs were expanded in cell medium until they were almost confluent. Noggin (500 ng/mL, R&D) and transforming growth factor-beta (TGF-β) inhibitor (10 mmol/L, Tocris) were added to confluent cells. The medium was replaced every 2 days with fresh KSR medium and different concentration gradients N2B27 medium. After nearly 10 days of differentiation, 100% N2 medium (DMEM/F12 supplemented with N2, GlutaMAX, EGF, FGF, heparin, penicillin, and streptomycin) was used to culture NSCs for one more day. Then, we transferred NSCs into a 100% N2/B27 medium (DMEM/F12 supplemented with N2, GlutaMAX, B27, FGF, EGF, heparin, penicillin and streptomycin).

### Plate coating

NSCs induction plates were coated with gelatin (Sigma) or Matrigel (BD). Human NSCs culture plates were precoated with poly-L-ornithine (Sigma) and laminin (Thermo Fisher). The 24-well plates and 6-well plates were freshly coated with gelatin or Matrigel incubating overnight at 4 °C to improve packing effects. The next day, the dishes were treated with 0.5 μg/mL poly-L-ornithine (dissolved in water) at room temperature for 16 h. Then, we washed the dishes with 1× phosphate-buffered saline (PBS). 5 μg/mL laminin was finally added to the dishes for at least 16 h. The coated dishes were stored at − 20 °C for future use, and the supernatant was discarded before use.

### Cell immunofluorescent staining

hGSCs were assessed using a staining assay, similar to that described in our previous study (Han et al. 2017). Briefly, hGSCs were cultured for 3 or 7 days in optimized culture conditions. Then, we fixed them in 4% paraformaldehyde (PFA) for 12 minutes. After cell fixation, 2.5% Triton X-100 in PBS were used to permeabilize cells with 15 minutes incubation. The supernatant was discarded for 1.5 hours cell blocking with 5% bovine serum albumin (Solarbio) in 1× PBS. All procedures were performed at room temperature. Primary antibodies including Sox2 (Goat, R&D), S100-β (Mouse, Abcam), or Ki67 (Rabbit, Thermo Fisher) were diluted as manufacturer protocol and added for 2 days incubation at 4 °C. Three times of wash were performed with 0.1% Tween-20 (Sigma) in 1× PBS. Secondary antibodies (Jackson Immuno Research), including Alexa Fluor 633-conjugated donkey anti-goat IgG antibody, Alexa Fluor 488-conjugated donkey anti-mouse IgG antibody, Alexa Fluor Cy3-conjugated donkey anti-goat IgG antibody, and Alexa Fluor 633-conjugated donkey anti-rabbit antibody were dissolved in PBS containing 2.5% bovine serum albumin. After 2 hours incubation at room temperature, three times of wash were performed with 0.1% Tween-20 (Sigma) in 1× PBS again. Finally, 4′, 6-diamidino-2-phenylindole (DAPI, Sigma) diluted with 0.1% Tween-20 in PBS was used to stain the nuclei. An inverted fluorescence microscope (Nikon TE2000) was used to obtain images of the immunofluorescent-stained cells.

### Sequencing and genomic analysis

We collected both hGSCs and hNSCs after 7 days of culture. After discarding the supernatant medium, 1× PBS was used to wash the cells once, and 2 ml Trizol (Invitrogen, Carlsbad, CA, USA) was added to each plate for RNA extraction. Agilent 2100 Bioanalyzer (Agilent, Palo Alto, CA, USA) was used to detect RNA integrity. Nanodrop (Thermo Fisher Scientific, Wilmington, DE, USA) was used to determine RNA quantity. Illumina TruSeqTM RNA sample preparation kit (Illumina Inc., San Diego, CA, USA) was used for fragmentation and cDNA synthesis priming. After that, ploy-T oligo-attached magnetic beads were used to purify poly-A containing mRNA molecules. All procedures were performed according to the manufacturer protocol. The cDNA was further converted into double-stranded DNA using the reagents supplied in the kit. AMpure XP beads were used to purify dsDNA. End-repaired and A-tailed were done according to Illumina’s protocol. PCR was applied to enrich the DNA fragments with adapter molecules on both ends and to amplify the amount of DNA in the library after adapter ligation. We pooled together the resulting molecular libraries and sequenced on a HiSeq 2500 sequencer (Illumina Inc.). According to the gene expression base, the fragments per kilobase of transcript per million mapped reads (FPKM) values were analyzed. Online software (Morpheus, https://software.broadinstitute.org/morpheus) was used to perform Differentially expressed gene (DEG) analysis. Online database (g:Profiler) (Reimand et al. 2007) was used for Kyoto Encyclopedia of Genes and Genomes (KEGG) and Gene Ontology (GO) analyses.

### Statistical analysis

All data were collected and analyzed based on three or more replicates. The error bars represent the standard deviation of the mean. Statistical analysis was performed using GraphPad Prism version 8.0.0 for Windows, GraphPad Software, San Diego, California USA, www.graphpad.comGraphPad Prism 7.0. For multiple comparisons, Student’s t-test was used to determine significant differences. Significance is indicated as *p < 0.05. **p < 0.01, and ***p < 0.001.

## Data Availability

The datasets used and/or analyzed during the current study are available from the corresponding author on reasonable request.

## Abbreviations

NSCs: Neural stem cells
GSCs: Glioma stem cells
IFN-β: Interferon-beta
hNSCs: Human NSCs
hGSCs: Human GSCs
CCNA2: Cyclin A2
CCNB1: Cyclin B1
CCNB2: Cyclin B1
CCND1: Cyclin D1
TME: Tumor microenvironment
JAK: Janus kinase–signal transducer
HBSS: Hank’s Balanced Salt Solution
DMEM: Dulbecco’s Modified Eagle Medium
MEF: Mouse embryonic fibroblast
TGF-β: Transforming growth factor-beta
PBS: Phosphate-buffered saline
PFA: Paraformaldehyde
FPKM: Fragments per kilobase of transcript per million mapped reads
DEG: Differentially expressed gene
KEGG: Kyoto Encyclopedia of Genes and Genomes
GO: Gene Ontology
BP: Biological process
CDK: Cyclin-dependent kinase
CD: Cytosine deaminase
5-FC: 5-fluorocytosine
